# CFTR Expression Analysis in Human Nasal Epithelial Cells by Flow Cytometry

**DOI:** 10.1371/journal.pone.0027658

**Published:** 2011-12-07

**Authors:** Marit A. van Meegen, Suzanne W. J. Terheggen-Lagro, Cornelis K. van der Ent, Jeffrey M. Beekman

**Affiliations:** 1 Department of Pediatric Pulmonology, University Medical Center Utrecht, Utrecht, The Netherlands; 2 Center for Molecular and Cellular Intervention, University Medical Center Utrecht, Utrecht, The Netherlands; University of Giessen Lung Center, Germany

## Abstract

**Rationale:**

Unbiased approaches that study aberrant protein expression in primary airway epithelial cells at single cell level may profoundly improve diagnosis and understanding of airway diseases. We here present a flow cytometric procedure to study CFTR expression in human primary nasal epithelial cells from patients with Cystic Fibrosis (CF). Our novel approach may be important in monitoring of therapeutic responses, and better understanding of CF disease at the molecular level.

**Objectives:**

Validation of a panel of CFTR-directed monoclonal antibodies for flow cytometry and CFTR expression analysis in nasal epithelial cells from healthy controls and CF patients.

**Methods:**

We analyzed CFTR expression in primary nasal epithelial cells at single cell level using flow cytometry. Nasal cells were stained for pan-Cytokeratin, E cadherin, and CD45 (to discriminate epithelial cells and leukocytes) in combination with intracellular staining of CFTR. Healthy individuals and CF patients were compared.

**Measurements and Main Results:**

We observed various cellular populations present in nasal brushings that expressed CFTR protein at different levels. Our data indicated that CF patients homozygous for F508del express varying levels of CFTR protein in nasal epithelial cells, although at a lower level than healthy controls.

**Conclusion:**

CFTR protein is expressed in CF patients harboring F508del mutations but at lower levels than in healthy controls. Multicolor flow cytometry of nasal cells is a relatively simple procedure to analyze the composition of cellular subpopulations and protein expression at single cell level.

## Introduction

Quantitative protein analysis at single cell level is critically important to study cell-type specific regulation of protein function in health and disease but limited techniques are available to perform single cell analysis in primary patient material [1], [2]. Flow cytometry has been widely used in immunology to study protein expression at single cell level of haematopoietic cells. The application of flow cytometry for other tissues is hampered by the ability to generate single cell suspensions, and the accessibility of patient samples.

Cystic fibrosis (CF) is caused by mutations of the gene encoding for Cystic Fibrosis transmembrane conductance regulator (CFTR) [3]–[5]. CF affects multiple organs but morbidity and mortality is dominated by CF lung disease that is characterized by mucus plugging, airway infections and sustained inflammation [6]. The most common mutation encodes for a CFTR protein that lacks phenylalanine at position 508 (F508del CFTR) causing it to misfold and retain in the endoplasmic reticulum from where is degraded [7], [8]. Contrasting data has been published on F508del CFTR protein expression levels in native airway epithelial cells. Kälin *et al.* showed endogenous wild type (wt) and F508del CFTR at similar intensity levels as healthy controls at the apical membrane in epithelial from nasal polyps [9]. This is in accordance with a study published by Penque *et al*. who observed apical CFTR in nasal epithelial cells from homozygous F508del patients, although it was found that the percentage of CFTR positive cells were significantly lower [10]. In addition Borthwick *et al.* recently reported similar CFTR expression at the apical surface between non-CF and CF cells in bronchial epithelium, although in CF cells the amount of CFTR expression was reduced [11]. However, Kreda *et al.* could not detect F508del CFTR at the apical membrane, and reported that the immature form of CFTR that resides in the ER was present at much lower levels [12], [13]. So quantification of CFTR protein expression has been proven difficult and it remains unclear whether differences in CFTR expression levels of individual patients can be related to residual function and CF disease variability in subjects harboring similar CFTR mutations.

Here, we developed a novel unbiased procedure to study CFTR expression in primary epithelial cells isolated from the nasal cavity at the single cell level by flow cytometry. CFTR function measurements in nasal epithelium correlate with CF disease indicating that nasal epithelium is a relevant tissue for studying CF disease mechanisms [14]. Nasal epithelial cells were harvested by a relatively non-invasive simple procedure. We validated this technique using other previously described techniques including Western blot analysis and RT-PCR. With this procedure we were able to study CFTR expression in nasal epithelial cells with a variety of CFTR antibodies, and found F508del homozygous patients to express CFTR protein, but at a lower level compared to healthy controls.

## Results

### Validation of CFTR expression in primary human epithelial cells

**Figure 1:**
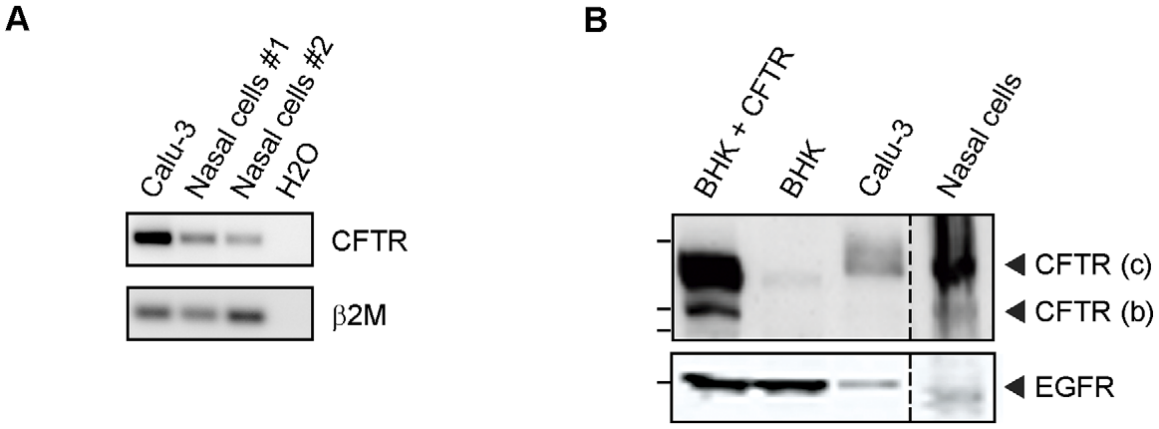
CFTR expression in cells obtained by nasal brushing. A. Quantitative real-time PCR analysis for CFTR and β2-microglobulin in nasal cells obtained from two healthy donors. Calu-3 cells were used as positive control. **B**. Western blot analysis of CFTR in brushed nasal cells. CFTR protein was detected as a mature form (c) and an immature form (b) with anti-CFTR mAb 24.1. Anti-EGF receptor was used as control.

Since CFTR expression analysis by Western blot in cells obtained from the nasal cavity by brushing is difficult due to contaminating cells and low cell yield we developed a novel assay to study CFTR protein level in individual nasal epithelial cells. However, we first validated that cells isolated by nasal brushing expressed CFTR as has been shown by others [10], [15], [16]. As expected, we observed CFTR mRNA expression in nasal cells obtained from two healthy individuals as indicated by RT-PCR (Fig. 1A). Calu-3 cells served as positive control and β2M was amplified as control for cDNA input. To assess CFTR protein expression in cells obtained from the nasal cavity, we pooled samples of multiple healthy individuals and performed Western blot analysis. We observed immunoreactivity around 170 kDa and to a lesser extent around 150 kDa in nasal cells and Calu-3 cells that co-migrated with ectopic CFTR expressed by BHK cells (Fig. 1B). This suggests that nasal cells collected by brushing expressed the fully glycosylated mature form (band C) and the immature form (band B) of CFTR. Together, these data demonstrate that our nasal brushings contained CFTR-expressing cells.

### Differentiation of nasal brushing cells by flow cytometry

**Figure 2:**
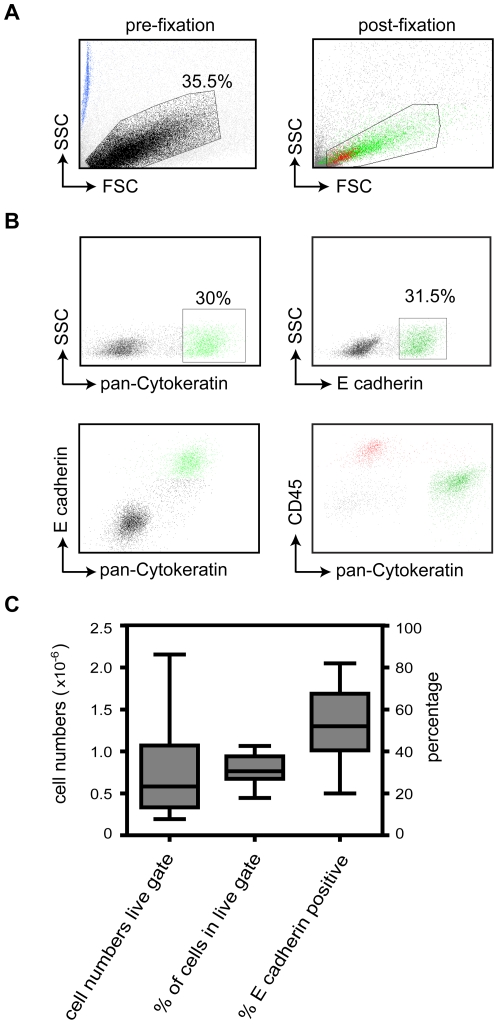
Analysis of nasal epithelial cells by flow cytometry. A. Forward and side scatter analysis (FSC and SSC) of brushed nasal cell before (left panel) or after fixation (right panel) by flow cytometry. Flow count beads (blue events, left panel) were used to count cells. In the right panel, epithelial cells are indicated by green events and haematopoietic cells by red events. **B.** Labeling of cellular subpopulations in the nasal cavity. Epithelial cells were stained using IgG1 mAb pan-Cytokeratin and IgG2a mAb E cadherin, and haematopoietic cells with CD45. **C**. Quantification of cell yield and cellular subpopulations in nasal brushing. Box-and-whiskers plot with five-number summaries, from 10 different healthy individuals were indicated for amount of events in the live gate (left bar), the percentage of live gate events (middle bar) and the percentage of E cadherin positive cells (right bar) within the live gate.

We next analyzed the yield and composition of cells isolated by brushing using flow cytometry. Single cell suspensions were generated by resuspending brushed cells in ice cold PBS containing EDTA and removing remaining cell clumps by passing cells through a 50 µM filter. Forward and side scatter (FSC and SSC) analysis by flow cytometry revealed a population of cells (gated in Fig. 2A) that was negative for propidium iodide (data not shown). To determine the cellular composition within this gated population, cells were stained intracellular for the epithelial markers pan-Cytokeratin and E cadherin or the haematopoietic marker CD45 (Fig. 2B). We could clearly distinguish E cadherin and pan-Cytokeratin double positive cells from CD45-expressing haematopoeitic cells that displayed a relative confined FSC/SSC profile. Considerable variation in cellular yield (counted using flowcount beads and indicated by blue events in the left panel of Fig. 2A) and composition was obtained between brushings (Fig. 2C). Gated events varied between 0.16 and 1.1×10^6^ events per brushing, and consisted of approximately 30% of total events. Within this gate, approximately 50% of cells expressed epithelial markers. In conclusion, these data indicate that epithelial cells from nasal brushings could be separated into single cell suspensions and distinguished from leucocytes using pan-Cytokeratin or E cadherin as marker by flow cytometry.

### Validation of CFTR antibodies for flow cytometry in cell lines

To demonstrate the capacity of various CFTR-directed monoclonal antibodies to recognize CFTR by flow cytometry, we used an identical procedure to process cell lines stably expressing human CFTR or non-transfected control cells. Intracellular staining for CFTR was performed using mAb 24.1, M3A7, L12B4, 217, 432, 450, 570, 596, and 769 on CFTR-BHK cells and non-transfected control cells. CFTR expression was indicated by mean fluorescence intensity (MFI) and CFTR-BHK cells were compared to non-transfected BHK cells for four different dilutions of the mAb. CFTR-specific detection was observed for mIgG1 432, 450, 570 and 769, mIgG2a 24.1 and mIgG2b 596 and only limited for mIgG_1_ M3A7. For mAbs 217 (mIgG1) and L12B4 (mIgG2a) high background levels were detected in non-transfected BHK and only a modest fluorescent increase was detected in CFTR-BHK cells. For L12B4 specific detection of CFTR was observed upon transient transfection of hCFTR in HEK293 cells, in contrast to mAb 217. We further confirmed specific detection of CFTR by mAb 450 in CFBE41ō cells transduced with wt-CFTR or F508del-CFTR.

**Figure 3:**
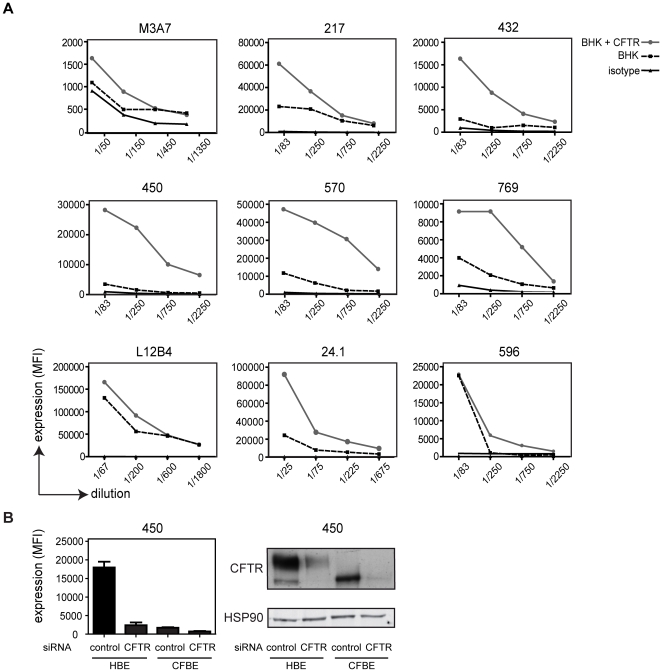
Validation of CFTR antibodies for flow cytometry using ectopically CFTR-expressing cells. A. A panel of nine monoclonal CFTR antibodies was used to stain CFTR in BHK cells ectopically expressing CFTR (grey line) or non-transfected control cells (black dotted line). Cells were stained intracellularly for CFTR, indicated by DyLight 649 conjugated goat anti-mouse antibody. Serial dilutions were performed and one of three representative experiments is shown. **B.** Paired analysis of CFTR detection by flow cytometry and Western blot using anti-CFTR mAb 450. Wild type CFTR or F508del–transduced CFBE41ō cells were transfected with CFTR siRNA or scrambled siRNA and evaluated for CFTR protein expression after 48 hours by flow cytometry (left panel; mean ± SEM; n = 3) and Western blot (right panel). HSP 90 was used as a loading control.

Knockdown of CFTR by siRNA in CFBE abolished CFTR detection both by flow cytometry and Western blot analysis (Fig. 3B). Similar data was observed using mAbs 24.1, 570, and 769 (see Figure S1). Together, these data indicate that ectopically expressed CFTR can be detected by flow cytometry using mAbs 432, 450, 570, 596, 769, L12B4 and 24.1 and only limited for mAb 217.

### CFTR detection in human nasal epithelial cells by flow cytometry

**Figure 4:**
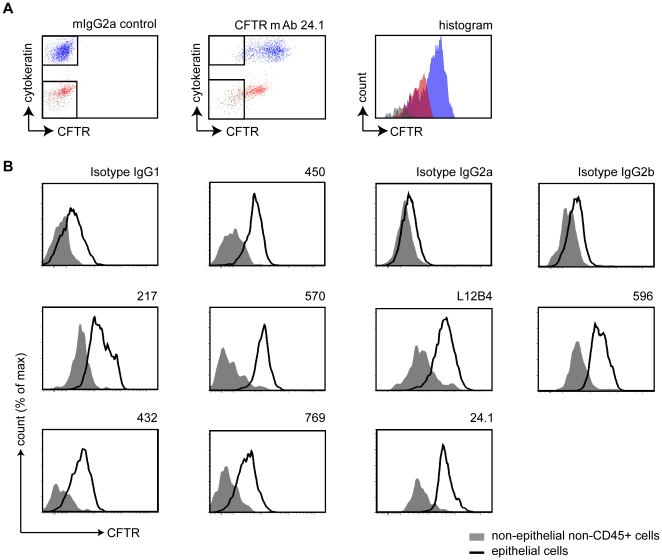
CFTR detection in human nasal epithelial cells by flow cytometry. A. Nasal cells were intracellularly stained using CFTR mAb 24.1 and pan-Cytokeratin and two subclass-specific secondary Abs conjugated to DyLight 488 and DyLight 649. CD45-Pecy7 staining was performed to visualize haematopoietic cells. The left panel shows isotype control staining for the CFTR mAb, the middle panel shows CFTR staining, and the right panel a histogram of CFTR staining intensities for non-pan-Cytokeratin non-CD45 cells (grey), CD45+ cells (red), and pan-Cytokeratin positive cells (blue). **B.** Intracellular staining for CFTR using multiple CFTR-directed mAbs. Histograms are shown that indicate CFTR staining intensity (or representative isotype control) of epithelial cells gated by E cadherin or pan-Cytokeratin (black line) and non-epithelial non-CD45 positive cells (grey). A representative example out of three is shown.

Subsequently, we studied CFTR expression by multi-color flow cytometry in cells from the nasal cavity using antibodies that were shown to detect CFTR in ectopic expression systems. Since we performed indirect stainings and used isotype-specific secondary antibodies, we combined mIgG1 anti pan-Cytokeratin with non-mIgG1 CFTR mAbs and mIgG2a anti-E cadherin with CFTR-specific mIgG1 mAbs. CFTR-directed mAbs selectively stained epithelial cells in contrast with isotype control staining. A representative staining of mAb 24.1 is indicated in Fig. 4A. Non-epithelial, non-CD45 cells, presumably stromal cells such as fibroblasts, displayed only limited reactivity. Some immunoreactivity of CFTR mAbs was observed for haematopoietic cells (CD45 positive). We also observed selective staining of epithelial cells using mAbs 432, 450, 570, 769, L12B4 and 596 (Fig. 4B). Differences in staining intensity of non-epithelial cells and epithelial cells were less pronounced for mAb 217 probably due to aspecific binding. These data suggest that mAb 432, 450, 570, 769, 24.1, L12B4 and 596 are suitable for CFTR detection in primary nasal epithelial cells by flow cytometry.

### Subcellular localization of CFTR in primary human nasal epithelial cells

**Figure 5:**
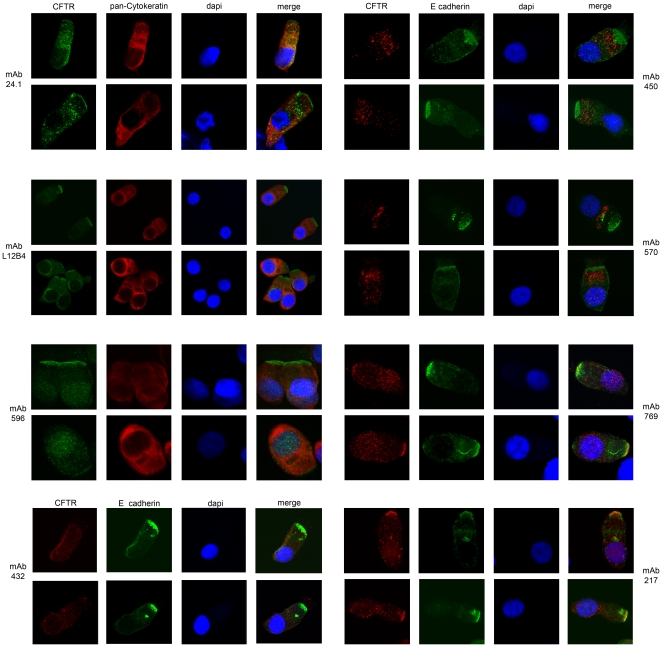
Immunolocalization of CFTR in nasal epithelial cells. Confocal analysis of CFTR in nasal epithelial cells of healthy individuals, two representative examples for all CFTR mAbs are shown. CFTR mIgG2 subclasses 24.1, L12B4, and 596 (green) were simultaneously stained with pan-Cytokeratin (red). CFTR mIgG1 subclass antibodies 217, 432, 450, 570 and 769 (red) were stained with E cadherin. Nuclei were indicated by dapi staining. The right panel shows a merge of all stainings.

To further confirm the staining specificity of the CFTR-directed mAbs in primary nasal epithelial cells of healthy individuals, we studied the localization of mAb staining by confocal microscopy. Staining patterns were analyzed in ciliated columnar epithelial cells that were selected by differential interference contrast microscopy. The epithelial origin of cells was indicated by co-staining with pan-Cytokeratin or E cadherin depending on the CFTR antibody subclass. Most CFTR antibodies were identified at the apical membrane and in intracellular organelles, albeit at variable levels. Two representative examples are indicated for each antibody in Fig. 5. Staining of the apical surface was detected for mAb 24.1, L12B4, 217, 596 and 769. MAbs 450 and 570 predominantly stained at intracellular compartments. Nuclear staining was also observed for mAbs 217, 432, 596 and 769 suggesting non-CFTR specific staining of a nuclear protein or contaminating mouse Ig since all CFFT mAbs are provided as diluted ascites fluid. We therefore excluded these antibodies from further experiments. Collectively, these data suggests that CFTR is localized at the apical membrane and intracellular compartments in nasal epithelial cells, and that different CFTR-specific antibodies preferentially bind to CFTR localized at distinct subcellular sites.

### CFTR expression in nasal epithelium of healthy individuals and CF patients

**Figure 6:**
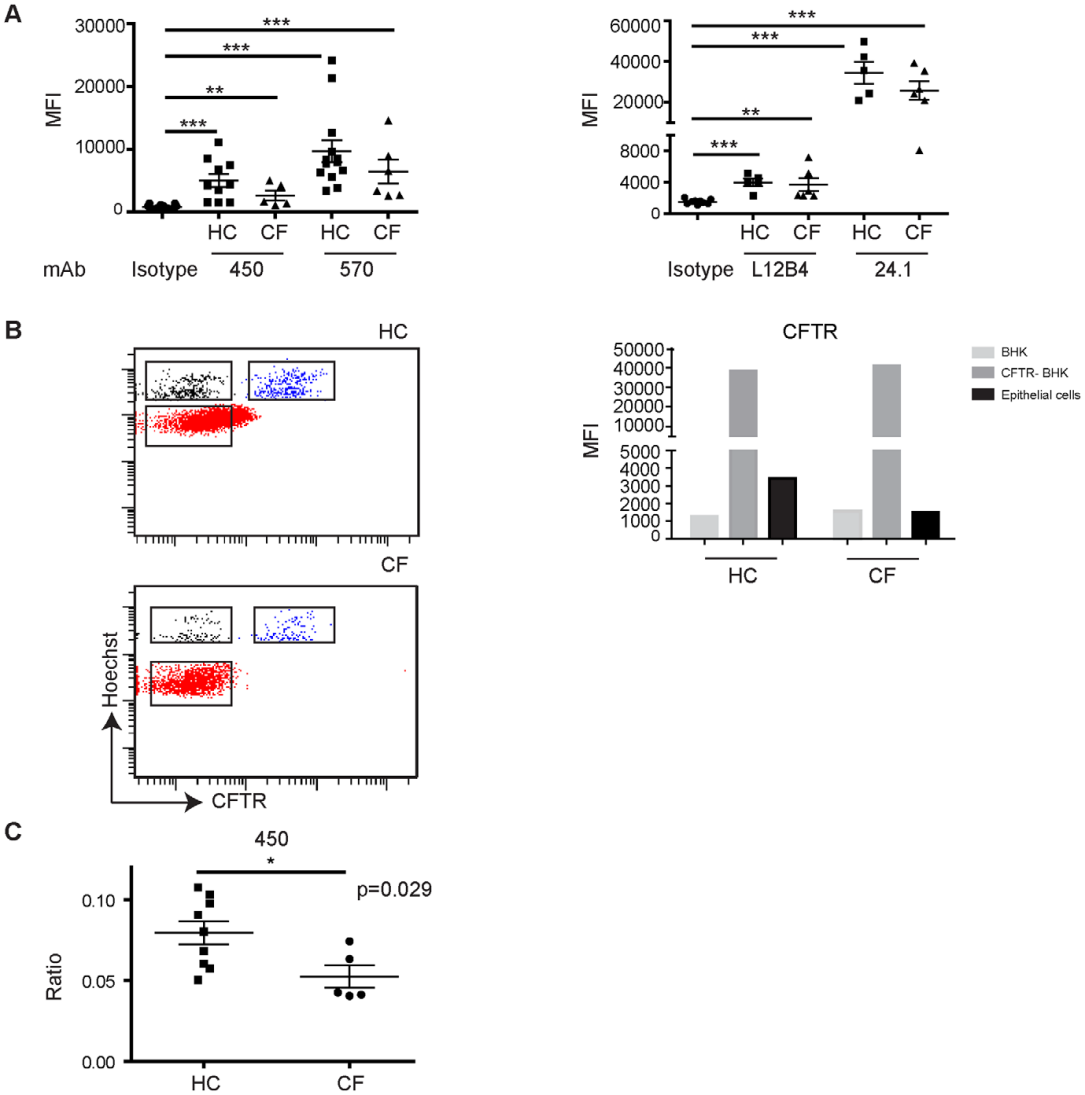
CFTR expression levels in nasal epithelial cells obtained from healthy individuals and CF patients. A . CFTR expression levels in nasal epithelial cells were assessed by flow cytometry with four CFTR mAbs of multiple healthy persons and CF patients. CFTR expression levels are indicated by mean fluorescent intensity (MFI). Staining with a mIgG1 CFTR mAb 450, 570 isotype control are indicated in the left panel, whereas mIgG2a CFTR mAbs L12B4 and 24.1 compared to isotype control are indicated in the right panel. Average ± SEM are shown. **B**. Representative example of CFTR staining on a healthy or CF individual including an internal standard. Aliquots of frozen BHK cells transfected with or without CFTR mixed at 1∶1 ratio, and pre-labeled with Hoechst were thawed and added to nasal epithelial cells prior to E cadherin and CFTR staining. Hoechst-positive CFTR-BHK cells (blue) and BHK cells (grey), and E cadherin positive cells (red) were gated and represented in dot plots showing Hoechst and CFTR staining (left panel) and represented in a histogram (right panel). **C.** Ratio of CFTR levels of E cadherin positive cells and CFTR-transfected BHK cells determined for 5 CF individuals and 9 healthy controls. Data represent average ± SEM.

To compare CFTR expression levels in healthy individuals and CF patients we used four CFTR antibodies that were selected based on their capacity to detect CFTR by flow cytometry in ectopic-CFTR expressing cells and nasal epithelium, and by low nuclear staining. For both healthy controls and CF patients we observed CFTR-specific staining of pan-Cytokeratin positive cells as indicated by isotype control staining (Fig. 6A). Somewhat reduced staining intensity was observed for CF patients using mAbs 450, 570 and 24.1 but no statistical differences were observed between MFI of healthy controls and CF cells upon staining with CFTR-directed antibodies. To better control for technical variability of CFTR staining intensity of brushings collected from different persons at different days, we added an internal control consisting of a small amount of BHK cells transfected with CFTR and non-transfected control BHK cells in a 1∶1 ratio to each sample. We selected mAb 450 to study CFTR based on its discriminatory power to detect ectopic CFTR in BHK cells (representative stainings for a healthy and CF individual are indicated in Fig. 6B). CFTR expression levels (MFI) were assessed by comparing the levels of the E cadherin positive cells with the CFTR-transfected BHK cells (Fig. 6C). Using this MFI we found significantly reduced CFTR staining in CF individuals as compared to healthy individuals. Collectively, our data suggest that CFTR is significantly expressed by CF patients (homozygous for the F508del mutation or with a F508del/ nonsense mutation) but at lower levels than healthy controls.

## Discussion

Here, we describe a novel procedure to study CFTR expression in small amounts of primary human nasal epithelial cells using flow cytometry. We demonstrated that CF patients express significant total levels of CFTR protein, although reduced compared to healthy controls. Flow cytometric analysis of relative CFTR expression levels offers important advantages over conventional approaches to study CFTR expression in patient materials. Thus far, most data has been generated by preparing cell lysates from which CFTR mRNA and protein is analyzed as we did in Fig. 1. Importantly, these techniques do not indicate the differences in CFTR expression of individual cells. In contrast, flow cytometric analysis allowed us to study the cellular composition of nasal brushings and relative CFTR expression levels of various subpopulations in an unbiased fashion (Fig. 2, 3, 4). Other advantages of flow cytometry over Western blotting are the low cell numbers required for analysis, short procedures, and the absence of enzymatic procedures to detect CFTR-bound antibodies. The considerable variation in the amount of leukocytes present in different nasal brushings also implies that CFTR expression analysis by Western blotting requires normalization for lower CFTR expressing cell subsets such as leukocytes. Furthermore, proteases present in leukocytes such as neutrophils can degrade CFTR in cell lysates, even in the presence of various protease inhibitor cocktails (data not shown).

Since CFTR detection by flow cytometry has not carefully been addressed in current literature, we compared a panel of nine monoclonal CFTR antibodies. Differences in CFTR localization for different antibodies observed by confocal microscopy could be explained by epitope blocking due to interacting proteins present at specific cellular compartments. Other- groups also investigated CFTR staining specificity of various CFTR-directed mAbs by immunofluorescence [9]–[11], [17]. In general, similar staining patterns could be observed. In contrast to previous studies we demonstrated L12B4 staining at the apical region instead of in intracellular structures similar in morphology and structure to the Golgi apparatus [17], [18]. This could be explained by the different fixation methods used and different antibody batches as we observed for L12B4. CFTR-specific antibodies preferentially bind to CFTR localized at distinct subcellular sites.

In our study we found that CFTR expression levels were lower in CF patients as compared to in healthy individuals and these differences reached statistical significance when an internal standard was used to correct for variation in staining intensity between brushings obtained at different days. These data are in concordance with earlier findings that indicated that considerable CFTR levels are expressed in airway epithelial cells in CF patients [9]–[11], [17], [19].

Flow cytometric analysis of CFTR can be used to study the relation between CFTR expression and patient-specific disease course. Considerable variation in staining intensity was observed within homozygous F508del patients that may associate with differences in residual CFTR function and clinical phenotype. Measurement of apically-located CFTR is presumably more important for associations between CFTR protein expression and clinical phenotype and may be developed using our procedure to generate single epithelial cells from brushings followed by sophisticated image analysis techniques. The generation of novel CFTR antibodies directed against extracellular epitopes would also be an important addition. Further optimization of this procedure may also be important for monitoring of CFTR-directed pharmacotherapy such as CFTR correctors, and identification of novel biomarkers. Furthermore, studying CFTR expression in different epithelial subsets using markers specific for ciliated, basal or goblet cells could be used to more selectively stain high CFTR expressing cells.

In conclusion, we developed a novel procedure to generate single cells from nasal brushings that can by analyzed by flow cytometry. It is a relatively quick and simple procedure and cell yields are sufficient to study protein expression levels of various subsets of cells present in the nasal cavity. Here we studied CFTR protein expression in healthy individuals and CF patients and found the latter to express lower levels of CFTR. In general, our procedure may be used to study protein expression in nasal epithelium in an unbiased fashion in patients that have altered airway epithelium such as asthma, chronic obstructive pulmonary diseases and cystic fibrosis.

## Materials and Methods

### Ethics statement

This study was conducted according to the principles expressed in the Declaration of Helsinki. The Study was approved by the Medical Research Ethics Committee of the UMC Utrecht, Ref 10-095. All participants provided written informed consent for the collection of samples and subsequent analysis.

### Cell lines

Calu-3 cells, a human lung adenocarcinoma cell line, endogenously expressing CFTR (ATCC, Manassas, VA) and baby hamster kidney cells (BHK, courtesy of Dr. Ineke Braakman, Utrecht University, The Netherlands) were grown in DMEM containing 10% fetal calf serum. CFTR-transfected BHK cells were grown in the presence of methotrexate 5 µM (Pharmachemie, Haarlem, The Netherlands). Human bronchial epithelial cells (CFBE41ō), derived from a CF patient and stably transduced with wild type CFTR or the ΔF508 mutation, were grown as described (courtesy of Dr. B.A. Stanton, Dartmouth Medical School, NH) [20].

All cell lines were cultured in media containing penicillin (100U/ml) and streptomycin (100 µg/ml) and kept in humidified incubators at 37°C containing 5% CO_2_. Cell culture media and reagents were obtained from Invitrogen (Carlsbad, CA).

### Human nasal epithelial cells

Nasal epithelial cells from healthy volunteers or CF patients were collected as described [16]. Cytological brushes were obtained from cell tip (Servoprax, Wesel, Germany). Cells were collected in DMEM and centrifuged at 800xg for 5 min, at 4°C. Thereafter resuspended in PBS and EDTA 5 mM for 15 minutes, and filtered through cup Filcons 50 µm (BD, San Jose, CA) to generate single cell suspensions. Cells were counted by flow cytometry using flow-count beads (Beckman-Coulter, Brea, CA).

### Primary antibodies

The following CFTR-specific antibodies were used: monoclonal antibody (mAb) 24-1 (mouse IgG2a) (R&D systems, Minneapolis, MN), mAb L12B4 (mIgG2a) (Chemicon international; Temecula, CA) and mAb M3A7 (mIgG1) (Chemicon International). The following antibodies were obtained via the Cystic Fibrosis Foundation Therapeutics (www.cftrfolding.org/CFFTReagents.htm) and were generously provided by Dr. J. Riordan (Department of Biochemistry and Biophysics and Cystic Fibrosis Center of North Carolina at Chapel Hill, NC); mAbs 217, 432, 450 and 570 (mIgG1) [12], [21]–[24], 596 (mIgG2b) [12], [21]–[24] and 769 mAb (mIgG1) [12], [24]. Other antibodies used in this study were mIgG1 mAb anti pan-Cytokeratin (Santa Cruz, CA), mAb mIgG2a anti-E cadherin (Beckton Dickinson, NJ) and FITC-conjugated mouse anti-EGF receptor (Becton Dickinson). Mouse anti-human CD45-PeCy7 (BD). The rabbit serum against Hsp90 was purchased from Ineke Braakman, Utrecht University, The Netherlands.

### RNA isolation, cDNA synthesis and real-time quantitative RT-PCR

RNA was extracted from nasal epithelial cells by RNAeasy columns (Qiagen, Hilden, Germany), according to the manufacturer's protocol. Thereafter total RNA was reverse-transcribed into cDNA using I script cDNA synthesis kit (Bio-Rad, Hemel Hempstead, UK). Real-time PCR was performed and quantified with a SYBR Green containing PCR mix (Bio-Rad). CFTR was amplified using forward primer GGACAGTTGTTGGCGGTTGC and reverse primer CTTGGAGATGTCCTCTTCTAG TTG as previous described [25]. Human β_2_-microglobulin (forward primer ATGAGTATGCCTGCCGTGTGA and reverse primer GGCATCTTCAAACCTCCATG) was amplified as control for cDNA synthesis and input. The PCR conditions were as follows: one cycle for 3 min at 95°C, 40 cycles of 30 sec at 95°C, 30 sec at 60°C and 30 sec at 72°C), 1 min at 95°C and for 1 min at 65°C. PCR products were analyzed using 1% agarose gel electrophoresis and ethidium bromide.

### Western blot analysis

Cell lysis, protein quantification and Western blot analysis was performed as described but with minor adaptations [26]. Collected nasal cells were lysed in Laemmli buffer in the presence of protease inhibitor cocktail (HALT, Thermo scientific, Rockford, IL), incubated for 30 min at 37°C, and passed through a 25G needle to reduce viscosity. Protein samples were quantified, separated on a 6% SDS-polyacrylamide gel, and transferred onto PVDF membrane. Membranes were blocked using milk protein, and probed with CFTR antibody 24.1 and EGFR as loading control. These were visualized by horseradish peroxidase-conjugated secondary antibody (Dako,Glostrup, Denmark), enhanced chemiluminiscent and films (Fuji Medical X-ray film, Tokyo, Japan).

### Intracellular staining

Cells were fixed with 50 µl Cytofix (BD pharmingen, San José, CA) per 5×10^4^ cells for 30 min at 4°C as all further steps. Next, cells were washed twice with Perm Wash buffer (BD) and incubated with 10% goat serum for 15 min in the same buffer. Subsequently, cells were incubated for 45 min with mIgG2a anti-E cadherin and mIgG1 anti-CFTR antibodies, or mIgG1 anti pan-Cytokeratin and mIgG2a/b anti CFTR for concentrations indicated. Cells were washed twice and incubated for 30 min with secondary antibodies at 1 µg/ml: goat anti-mouse IgG1 Dylight 649-conjugated and goat anti- mouse IgG2a Dylight 488 (Jackson Immuno Research, West Grove, PA). Cells were washed in Perm Wash buffer and resuspended in PBS containing 0.2% FCS and 0.1% sodium azide. Subsequently, cells were analyzed by flow cytometry using a FACS Canto (BD) or confocal microscopy using a Zeiss LSM710 and 63x objective (Zeiss, Heidelberg, Germany) after spinning cells on microscope glasses and embedding in Mowiol containing Dapi (4′,6-diamidino-2-phenylindole, Sigma) as described [27].

### CFTR Knockdown experiment

Four pre- designed siRNAs for CFTR were purchased (#1 GAACACAUACCUUCGAUAU; #2GUACAAACAUGGUAUGACU; #3GUGAAAGAC UUGUGAUUAC; #4GCAGGUGGGAUUCUUAAUA) (Thermo Fisher scientific, Lafayette, CO). Transfection with a non-targeting pool of siRNA (Thermo Fisher Scientific) was used as control. CFBE41ō cells stably transduced with either WT-CFTR or F508del CFTR were seeded in 10 cm^2^ dishes and transfected at 60% confluence with 20 nM siRNA using Lipofectamine RNAiMAX (Invitrogen) according to the manufacturer's instructions. CFTR analysis by flow cytometry and Western blot was performed 48h post transfection.

### Statistical analysis

Statistical analysis was assessed using a Student's t-test, and significance was accepted at p<0.05. All statistical analyzes were performed using Prism statistical software version 5 (Graph Pad, La Jolla, CA).

## Supporting Information

Figure S1 **Validation of the CFTR antibodies for flow cytometry in a cell line.** Paired analysis of CFTR detection by flow cytometry and Western blot using anti-CFTR mAbs 570, 769 and 24.1. Wild type CFTR or F508del–transduced CFBE41ō cells were transfected with CFTR siRNA or scrambled siRNA and evaluated for CFTR protein expression after 48 hours by (A) flow cytometry (mean ± SEM; n = 3) and (B) Western blot. HSP 90 was used as a loading control.
(TIF)
