# Supplementary figures and images for: CFTR Expression Analysis in Human Nasal Epithelial Cells by Flow Cytometry
Source: PLoS One. 2011 Dec 7;6(12):e27658. doi: 10.1371/journal.pone.0027658 (PMC3233544; doi:10.1371/journal.pone.0027658)

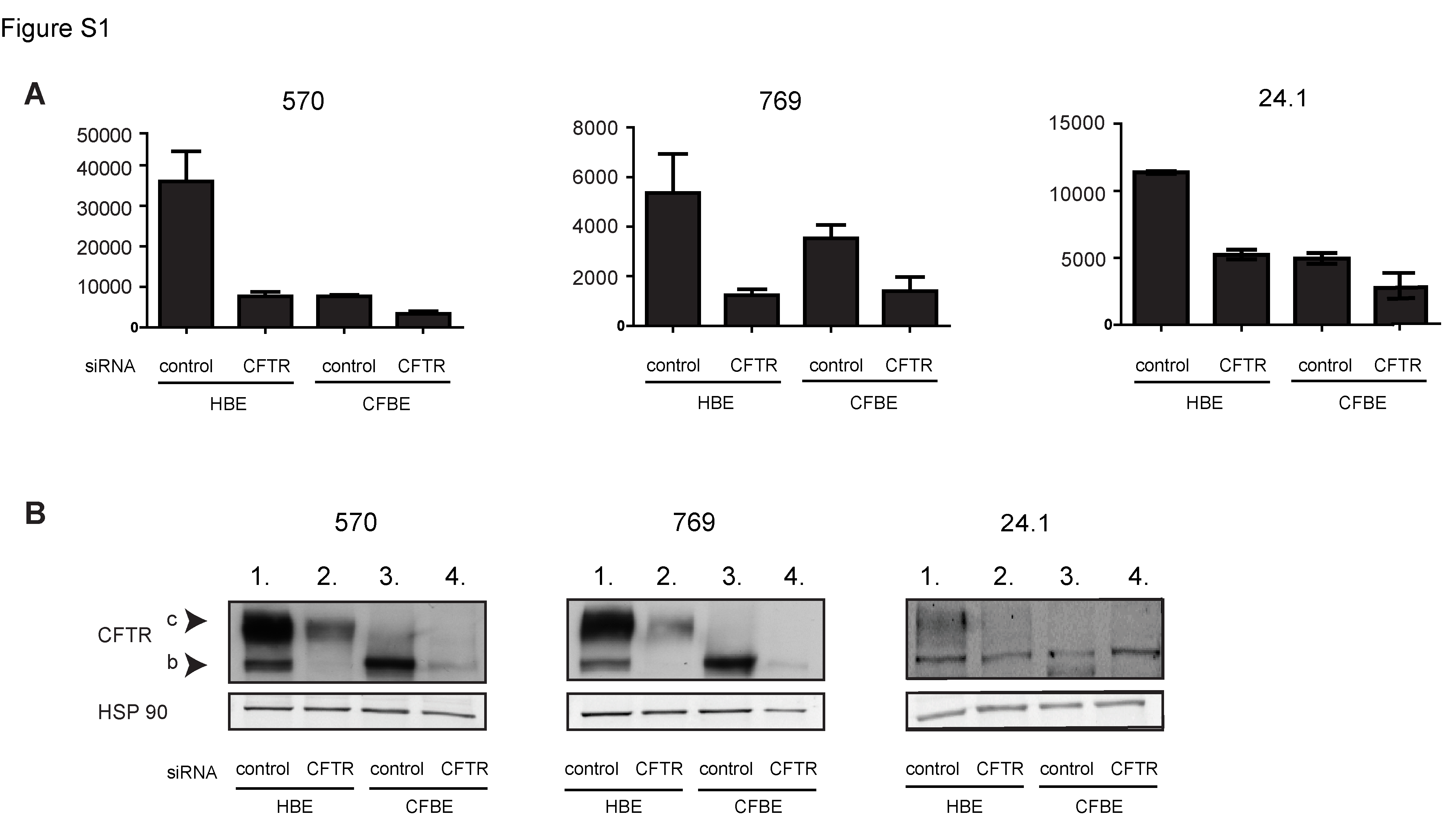

Supplement: Figure S1 — Validation of the CFTR antibodies for flow cytometry in a cell line. Paired analysis of CFTR detection by flow cytometry and Western blot using anti-CFTR mAbs 570, 769 and 24.1. Wild type CFTR or F508del–transduced CFBE41ō cells were transfected with CFTR siRNA or scrambled siRNA and evaluated for CFTR protein expression after 48 hours by (A) flow cytometry (mean ± SEM; n = 3) and (B) Western blot. HSP 90 was used as a loading control. (TIF) [file pone.0027658.s001.tif]
